# *PIK3CA* gene mutations in the helical domain correlate with high tumor mutation burden and poor prognosis in metastatic breast carcinomas with late-line therapies

**DOI:** 10.18632/aging.102701

**Published:** 2020-01-24

**Authors:** Yu Tang, Jing Li, Ning Xie, Xiaohong Yang, Liping Liu, Hui Wu, Can Tian, Ying He, Xiao Wang, Qiongzhi He, Zhe-Yu Hu, Quchang Ouyang

**Affiliations:** 1 The Affiliated Cancer Hospital of Xiangya School of Medicine, Central South University, Hunan Cancer Hospital, Changsha 410000, China; 2 Department of Breast Cancer Medical Oncology, Hunan Cancer Hospital, Changsha 410000, China; 3 The 2nd Department of Breast Cancer Surgical Oncology, Hunan Cancer Hospital, Changsha 410000, China; 4 ICF, Atlanta, GA 30329, USA; 5 Beijing Geneplus Institute, Beijing 102200, China

**Keywords:** metastatic breast cancers, PIK3CA mutations, helical domain, tumor mutation burden, ctDNA mutation profile

## Abstract

Nearly half of metastatic breast cancers (MBC) have genetic aberrations in the PI3K/AKT pathway. To investigate the distinct effect of these aberrations on MBC, 193 MBC patients who progressed after the early line (≤2) salvage treatment voluntarily received next generation sequencing (NGS) for a panel of 1,021 genes. 93 (48%) patients had genetic aberrations in the PI3K/AKT pathway. The number of patients with *PIK3CA* mutations in kinase domain (KD), helical domain (HD) and other domain (OD), were 36 (18.7%), 26 (13.5%), 10 (5.2%), respectively. 21 (10.9%) patients had mutations in PI3K/AKT pathway genes other than *PIK3CA* (P/A). Compared to PI3K/AKT-wild type (WT) patients, *PIK3CA*-HD patients had a significantly shorter progression-free survival (PFS) (Logrank *p*-value < 0.0001). *PIK3CA*-KD, *PIK3CA*-OD and other P/A mutations showed similar PFS to WT patients (Logrank *p*-value = 0.63). *PIK3CA*-HD patients had a distinct ctDNA mutation profile to patients with other PI3K/AKT mutations. *PIK3CA*-HD patients had a higher rate of *FGFR* and *NF1* aberrations. In addition, more *PIK3CA*-HD carriers were TMB-high. Cox regression analyses suggested that *PIK3CA*-HD mutations, *FGFR* aberrations and high TMB were all significant risk factors for poor PFS. In conclusion, future research needs to focus more on the treatment strategies targeting *PIK3CA*-HD mutations.

## INTRODUCTION

A recent study suggested *PIK3CA* mutations to be a major mediator of therapy resistance in breast cancer [1]. More than 70% of hormone receptor (HR)-positive breast cancers have molecular aberrations in Phosphatidylinositol 3-kinase (PI3K)-AKT-mTOR pathways [2]. PI3K is a heterodimer composed of a regulatory subunit p85 and a catalytic subunit p110 [3]. The *PIK3CA* gene encodes the PI3K catalytic subunit p110α [4]. According to the circulating tumor DNA (ctDNA) sequencing results, about 50% of HR-positive metastatic breast cancers (MBCs) have *PIK3CA* missense mutations; 10-30% of metastatic triple-negative breast cancers (TNBC) and HER2-positive breast cancers have *PIK3CA* missense mutations [5]. About 80% of *PIK3CA* mutations occur in helix domain (HD) exon 9 and kinase domain (KD) exon 20 [6]. 26% of *PIK3CA* mutations are in exon 9 (hotspots: E545K and E542K), and 50% of *PIK3CA* mutations are in exon 20 (hotspot: H1047R) [7]. While *PIK3CA-*HD mutations are less potent in inducing mammary tumors [8], these mutations are independently associated with poor prognosis (early recurrence and death) [9]. Although *PIK3CA*-KD mutations are associated with lymph node infiltration and are much more aggressive in carcinogenesis compared to *PIK3CA-*HD mutations [8, 10–11], *PIK3CA*-KD mutations are associated with optimal prognosis [9].

*PIK3CA-*HD mutations are more frequent in older age-onset patients, and they are not associated with lymph node infiltration as *PIK3CA*-KD mutations [10–11]. Importantly, the therapeutic response of *PIK3CA-*HD mutant tumors is significantly poorer than tumors with *PIK3CA*-KD mutations [12]. Mechanism studies suggest that, instead of binding to the p85 subunit, *PIK3CA-*HD mutation-encoded protein PIK3CA^(E545K)^ interacts with Ras-GTP [13]. Compared to PIK3CA^(H1047R)^, PIK3CA^(E545K)^ is less efficient in activating the downstream Akt signaling [13].

Most MBC patients receive chemotherapy coupled with/without endocrine therapy or anti-HER2 therapy. The activation of PI3K/AKT/mTOR pathway, however, would promote tumor progression and induce drug resistance to endocrine therapy and chemotherapy [14–16]. In this study, we examined the genetic aberrations of PI3K/AKT pathway and assessed the effect of these aberrations on progression-free survival (PFS) in metastatic breast cancer (MBC) patients with late-line treatment. We analyzed the ctDNA mutation profile and investigated the association of PI3K/AKT pathway aberrations with the clinical and genetic features of MBC tumors. We compared distinct types of PI3K/AKT pathway mutations, especially *PIK3CA*-HD and *PIK3CA*-KD mutations, to determine the different effects of PI3K/AKT pathway mutations on the prognosis of MBC patients with late-line treatment.

## RESULTS

### Genetic aberrations of PI3K/AKT pathway molecules in MBCs after early-line salvage therapy

**Figure 1:**
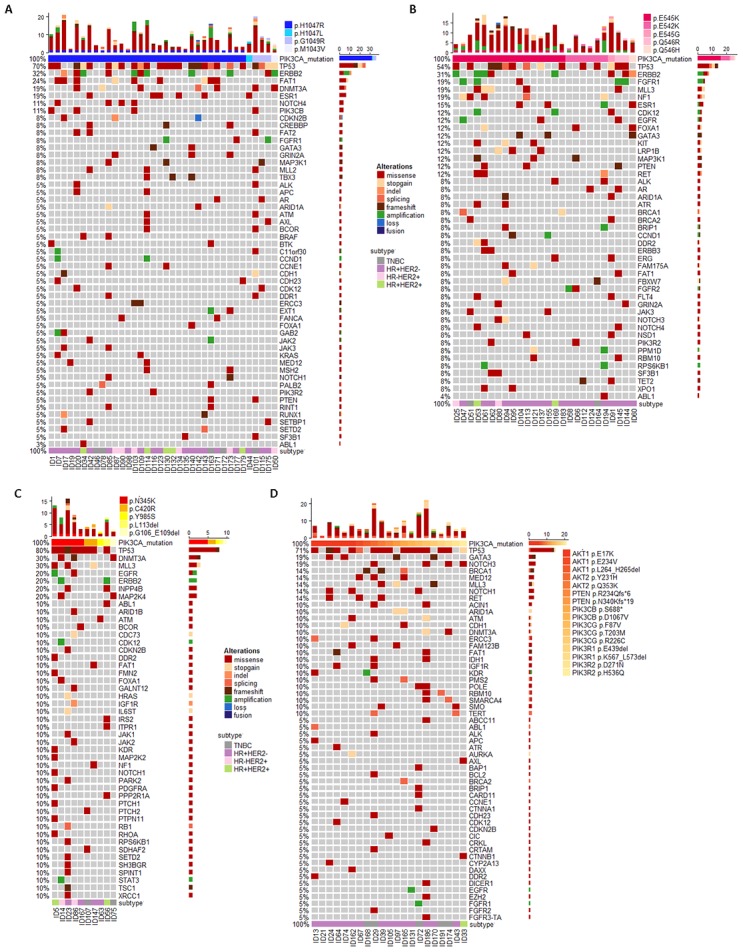
Circulating tumor DNA (ctDNA) gene mutation profiles for MBC patients who progressed after early-line therapy and had *PIK3CA* mutations in kinase domain (*PIK3CA*–KD, **A**) helix domain (*PIK3CA*–HD, **B**), other region (*PIK3CA*–OD, **C**) and other PI3K/AKT pathway aberrations (P/A, **D**).

A total of 193 MBC patients after 1^st^ or 2^nd^ salvage treatment received ctDNA testing and then late-line therapy between December 2016 and December 2018. Ninety three patients (48%) had ctDNA mutations of the PI3K/AKT pathway molecules. Among these ninety three patients, thirty six patients (38.7%) had *PIK3CA*–KD missense mutations, including thirty one p.H1047R, one p.H1047L, two p.G1049R, and one p.M1043V missense mutations (Figure 1A). Twenty six patients (28%) had *PIK3CA*–HD missense mutations, including sixteen p.H545K, six p.H542K, one p.E545G, two p.Q546R and one p.Q546H missense mutations (Figure 1B). Ten patients (10.8%) had *PIK3CA*-OD aberrations, including five p.N345K, two p.C420R and one p.Y985S missense mutation, one p.L113del and one p.G106_E109del mutation (Figure 1C). The other twenty one patients (22.6%) had aberrations in other PI3K/AKT pathway molecules except *PIK3CA*, including six *AKT1* p.E17K missense mutations, two other *AKT1* mutations, two *AKT2* mutations, two *PTEN* frameshifts, two *PIK3CB*, three *PIK3CG*, two *PIK3R1* and two *PIK3R2* mutations (Figure 1D).

Clinical features were distinct with respect to different PI3K/AKT aberrations (Table 1). Compared to the wild-type (WT) patients, patients with PI3K/AKT pathway aberrations had longer time from BC diagnosis to metastasis (TTM) (p = 0.001). More PI3K/AKT pathway aberrant patients had visceral metastases, and more PI3K/AKT pathway aberrant patients were ER-positive or PR-positive. In addition, compared to *PIK3CA*-KD mutant patients, *PIK3CA*-HD carriers had an even higher ER/PR-positive rate.

**Table 1:** Clinical characteristics of MBC patients with wild-type *PIK3CA* and PI3K/AKT pathways aberrations.

**Variables**	**Levels**	**WT (n=100)**	**PI3K/AKT pathway aberrations**				p-value*†	p-value**†
			***PIK3CA*-KD (n=36)**	***PIK3CA*-HD (n=26)**	***PIK3CA*-OD (n=10)**	**P/A (n=21)**		
Age at diagnosis (years)		43.7 ± 8.9, 42.9 (36.3, 49.8)	45.0 ± 9.5, 42.9 (37.9, 51.6)	45.9 ± 9.9, 44.6 (38.1, 51.0)	45.0 ± 11.4, 47.0 (37.6, 55.3)	41.7 ± 11.4, 40.0 (35.1, 52.0)	0.55	0.70
Age at metastasis (years) ^#^		46.4 ± 9.2, 46.2 (38.8, 52.3)	49.3 ± 9.3, 50.1 (41.9, 56.4)	51.2 ± 9.8, 52.5 (40.3, 56.4)	48.7 ± 12.1, 54.0 (38.0, 57.3)	44.4 ± 10.8, 42.2 (37.2, 52.3)	0.09	0.45
TTM ^##^		2.6 ± 2.8, 1.6 (0.3, 4.3)	4.4 ± 4.3, 2.8 (1.5, 5.4)	5.1 ± 3.6, 5.0 (3.4, 6.4)	3.7 ± 4.7, 2.3 (0.4, 3.0)	3.3 ± 2.9, 2.2 (0.6, 5.9)	0.001	0.53
ER	Positive	40 (40.0%)	22 (61.1%)	21 (80.8%)	5 (50.0%)	16 (76.2%)	0.0003	0.10
	Negative	60 (60.0%)	14 (38.9%)	5 (19.2%)	5 (50.0%)	5 (23.8%)		
PR	Positive	33 (33.0%)	19 (52.8%)	19 (73.1%)	6 (60.0%)	13 (61.9%)	<0.0001	0.10
	Negative	67 (67.0%)	17 (47.2%)	7 (26.9%)	4 (40.0%)	8 (38.1%)		
HER2	Positive	30 (30.0%)	11 (30.6%)	4 (15.4%)	4 (40.0%)	1 (4.8%)	0.21	0.23
	Negative	70 (70.0%)	25 (69.4%)	22 (84.6%)	6 (60.0%)	20 (95.2%)		
HR/HER2 subtype	TNBC	36 (36.0%)	5 (13.9%)	2 (7.7%)	2 (20.0%)	5 (23.8%)	0.27	0.46
	HR+/HER2-	34 (34.0%)	20 (55.6%)	20 (76.9%)	4 (40.0%)	15 (71.4%)		
	HR-/HER2+	21 (21.0%)	6 (16.7%)	2 (7.7%)	2 (20.0%)	0 (0%)		
	HR+/HER2+	9 (9.0%)	5 (13.9%)	2 (7.7%)	2 (20.0%)	1 (4.8%)		
Metastasis sites ^###^	Bone-only	16 (16.0%)	2 (5.6%)	4 (15.4%)	2 (20.0%)	4 (19.1%)	0.54	0.22
	Visceral	47 (47.0%)	28 (77.8%)	18 (69.2%)	5 (50.0%)	14 (66.7%)	0.001	0.45
	Soft tissue	53 (53.0%)	26 (72.2%)	16 (61.5%)	3 (30.0%)	12 (57.1%)	0.25	0.37

Note:

Patients with PI3K/AKT pathway aberrations were divided into four groups: WT (wild-type group), *PIK3CA*-KD (*PIK3CA* kinase-domain mutation) group, *PIK3CA*-HD (*PIK3CA* helix-domain mutation) group, *PIK3CA*-OD (other *PIK3CA* mutation) group, and P/A (other PI3K/AKT pathway mutations) group.

p-values† were calculated using Student’s *t*-tests for continuous variables and using Chi-square tests (Mentel-Haenszel for >2 levels comparison), or Fisher’s exact tests (n<5) for categorical variables

p-values* compared variables between wild-type patients and *PI3K/AKT* aberrant patients; p-value** compared variables between *PIK3CA*-KD mutant patients and *PIK3CA*-HD mutant patients.

Age at metastasis ^#^ represented the age (in year) of patients when the metastasis occurred. TTM ^##^ represented the time (in year) from diagnosis to metastasis.

Metastatic sites ^###^ compared the patients with bone-only metastases, visceral metastases (such as liver, lung, brain, ovary, etc), and soft tissue metastases (lymphnode, mediastinum, plura and contra-lateral breast).

Abbreviation: ER (Estrogen Receptor), PR (Progesterone Receptor), HER2 (Human Epidermal Growth Factor Receptor-2).

### Poor prognosis with *PIK3CA* mutations in helical domain

**Figure 2:**
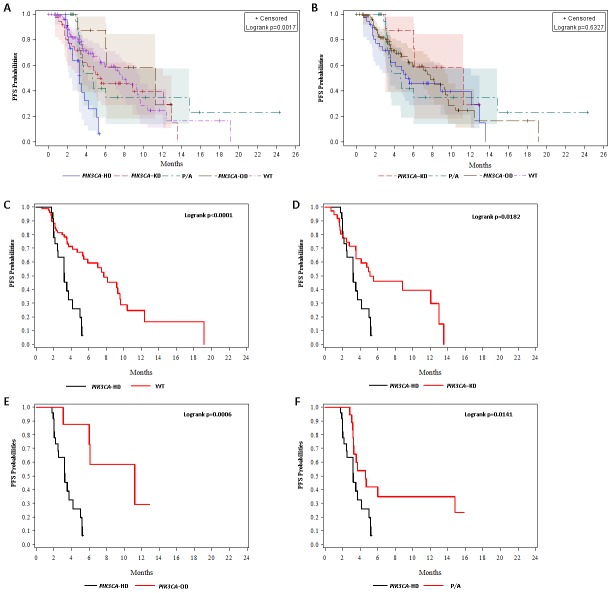
**Kaplan-Meier (KM) curves for progression-free survival (PFS) probabilities.** (**A**) KM curves for PFS probabilities stratified by wild-type (WT) and *PIK3CA*–KD mutations, *PIK3CA*–HD mutations, *PIK3CA*–OD mutations, and other PI3K/AKT pathway aberrations (P/A). (**B**) KM curves for PFS probabilities stratified by wild-type (WT) and *PIK3CA*–KD mutations, *PIK3CA*–OD mutations, and other PI3K/AKT pathway aberrations (P/A). (**C**) KM curves for PFS probabilities stratified stratified by wild-type (WT) and *PIK3CA*–HD mutations. (**D**) KM curves for PFS probabilities stratified stratified by *PIK3CA*–HD mutations and *PIK3CA*–KD mutations. (**E**) KM curves for PFS probabilities stratified by *PIK3CA*–HD mutations and *PIK3CA*–OD mutations. (**F**) KM curves for PFS probabilities stratified by *PIK3CA*–HD mutations and *other* PI3K/AKT pathway aberrations (P/A).

All included patients failed and progressed in the early–line (≤2) salvage therapy. Then, the later line regimen was given by TPC (treatment physician choice). The median PFS for patients with WT, *PIK3CA*-KD, *PIK3CA*-HD, *PIK3CA*-OD, other *PI3K/AKT* (P/A) mutations were 7.7 months (95% CI: 5.4-9.6), 5.1 months (95% CI: 3.5-13.0), 3.2 months (95% CI: 2.1-4.2), 11.2 months (95% CI: 3.0-NE), and 4.6 months (95% CI: 3.3-14.8), respectively (Figure 2). Apart from *PIK3CA*-HD mutations, patients with PI3K/AKT pathway aberrations had similar PFS to WT patients (Logrank p=0.63). *PIK3CA*-HD mutant patients had a significantly lower PFS than WT patients (Logrank p<0.0001), *PIK3CA*-KD mutant patients (Logrank p=0.02), *PIK3CA*-OD mutant patients (Logrank p=0.0006) and P/A aberrant patients (Logrank p=0.01).

### ctDNA mutation profile

Due to the metastatic tumor burden, we could detect ctDNA mutations in most MBC patients [5]. Compared to WT patients, patients with PI3K/AKT pathway aberrations had significantly higher rates of *TP53*, *ERBB2*, *FAT1*, and *FGFR* aberrations (Table 2). In addition, patients with *PIK3CA*-HD mutations had an obviously distinct ctDNA mutation profile (Figure 1). For example, compared to the *PIK3CA*-KD mutations, *PIK3CA*-HD mutant patients had a higher *FGFR* aberration rate (Fisher’s exact test, p=0.10), a higher *NF1* mutation rate (Fisher’s exact test, p=0.07), and a lower *DNMT3A* mutation rate (Fisher’s exact test, p=0.04) (Table 2).

**Table 2:** Somatic mutations accompanied with PI3K/AKT pathway gene aberrations.

**Variables**	**WT (n=100)**	**PI3K/AKT pathway mutations#**				p-value*†	p-value**†
		***PIK3CA*-KD (n=36)**	***PIK3CA*-HD (n=26)**	***PIK3CA*-OD (n=10)**	**P/A (n=21)**		
*TP53*	33 (32.0%)	26 (72.2%)	14 (53.9%)	9 (90.0%)	14 (66.7%)	<0.0001	0.14
*ERBB2*	8 (8.0%)	10 (27.8%)	8 (30.8%)	2 (20.0%)	0 (0%)	0.008	0.80
*FAT1*	3 (3.0%)	9 (25.0%)	2 (7.7%)	1 (10.0%)	2 (9.5%)	0.004	0.10
*ESR1*	5 (5.0%)	6 (16.7%)	4 (15.4%)	0 (0%)	0 (0%)	0.18	0.90
*DNMT3A*	6 (6.0%)	7 (19.4%)	0 (0%)	2 (20.0%)	2 (9.5%)	0.15	0.04
*FGFR*	7 (7.0%)	4 (11.1%)	8 (36.4%)	0 (0%)	3 (14.3%)	0.05	0.10
*NF1*	4 (4.0%)	1 (2.8%)	5 (19.2%)	1 (10.0%)	0 (0%)	0.36	0.07
TMB-High	5 (5.0%)	8 (22.2%)	14 (53.9%)	3 (30.0%)	4 (19.1%)	<0.0001	0.01

#Patients with PI3K/AKT pathway aberrations were divided into four groups: WT group, *PIK3CA-*KD group, *PIK3CA*-HD group, *PIK3CA*-OD group, and P/A (other PI3K/AKT pathway mutations) group.

p-values† were calculated using Student’s *t*-tests for continuous variables and using Chi-square tests (Mentel-Haenszel for >2 levels comparison), or Fisher’s exact tests (n<5) for categorical variables.

p-values* compared variables between WT patients and PI3K/AKT pathway aberrant patients; p-value** compared variables between *PIK3CA*-KD mutant patients and *PIK3CA*-HD mutant patients.

### High TMB in helical domain mutant patients

**Figure 3:**
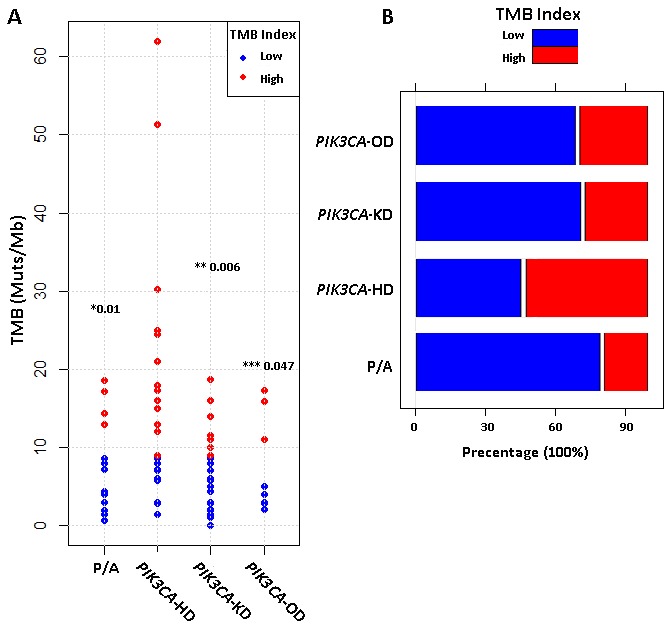
**Tumor mutation burden (TMB) for different metastatic breast cancer subtypes.** (**A**) Comparison of TMB levels (mutations per million bases, muts/Mb) among four types of PI3K/AKT pathway aberrations. p-value for *PIK3CA*-HD *vs PIK3CA*-KD (*), *PIK3CA*-HD *vs PIK3CA*-OD (**) and *PIK3CA*-HD *vs* P/A (***) were calculated by using non-parametric wilcoxon rank-sum test. (**B**) Barplot compared the percentages of TMB-High (red) and TMB-Low (blue) patients among four different types of PI3K/AKT pathway aberrations.

Tumor mutation burden (TMB) has been considered as a marker for immunotherapy. The more mutations the tumor has, the higher possibility of production and subsequent presentation of tumor-associated antigens (TAA) on MHC molecules, which leads to a higher tumor cell cytotoxicity after the inhibition of immune checkpoint signals [17, 18]. BCs are “cold” tumors with less TAAs than “hot” tumors (non-small cell lung cancer and malignant melanoma) [19, 20]. In this study, we defined TMB-H if the TMB was larger than the top 25% TMB value of all BC samples in Geneplus database (9 Muts/Mb). This cut-off value of 9 Muts/Mb was reasonable because it was equal to the lung cancer of 9 Muts/Mb (the third third tertile) [21] and close to the gastric cancer of 12 Muts/Mb (top 20%) [22]. Here, we found that *PIK3CA*-HD mutant MBC patients had a significantly higher TMB level than patients with *PIK3CA*-KD mutations (p=0.006), *PIK3CA*-OD mutations (p=0.045), or other P/A pathway mutations (p=0.01) (Figure 3).

### Risk prognostic factors for MBC patients with late-line therapy

Cox regression analyses suggested that none of the clinical indicators had significant impacts on PFS for late-line therapies, including the age of diagnosis, age of BC metastasis, TTM, primary BC laterality, ER, PR, HER2 status and the sites of metastatic lesions (Supplementary Table 1). However, we found that some genetic indicators, including *PIK3CA*-HD mutations, *FGFR* aberrations, and high TMB levels, were significant risk factors for poor PFS for late-line therapy in MBCs (Table 3). Multivariate Cox regression analyses showed that compared to PI3K/AKT pathway WT MBCs, the hazard ratios (HR) for patients with *PIK3CA*-HD mutations, *TP53* mutations, *FGFR* aberrations, and high TMB levels were 2.0 (95% CI = 1.02-3.93, p=0.045), 2.22 (95% CI = 1.35-3.66, p=0.0002), 2.17 (95% CI = 1.17-4.02, p=0.01), and 1.62 (95% CI = 1.00-2.66, p=0.05), respectively.

**Table 3:** Cox regression analyses of genetic risk factors for PFS in MBC patients.

**Covariates**	**Level**	**Univariate Cox Model**			**Multivariate Cox Model**	
		**HR (95% CI)**	**p-value**		**HR (95% CI)**	**p-value**
PI3K/AKT pathway	WT	Ref			Ref	
mutations	*PIK3CA*-HD	2.99 (1.64, 5.44)	0.0004		2.00 (1.02, 3.93)	0.045
	*PIK3CA*-KD	1.22 (0.72, 2.09)	0.45		0.75 (0.39, 1.42)	0.37
	*PIK3CA*-OD	0.64 (0.23, 1.80)	0.39		0.40 (0.14, 1.18)	0.10
	P/A	0.95 (0.49, 1.85)	0.88		0.80 (0.40, 1.57)	0.51
*TP53* aberration	No	Ref			Ref	
	Yes	2.25 (1.43, 3.54)	0.0004		2.22 (1.35, 3.66)	0.002
*ERBB2* aberration	No	Ref			Ref	
	Yes	1.54 (0.93, 2.55)	0.09		1.45 (0.80, 2.65)	0.23
*FAT1* aberration	No	Ref			Ref	
	Yes	2.06 (1.06, 4.00)	0.03		1.48 (0.74, 2.98)	0.27
*ESR1* aberration	No	Ref			Ref	
	Yes	1.16 (0.56, 2.43)	0.69		1.25 (0.58, 2.71)	0.60
*DNMT3A* aberration	No	Ref			Ref	
	Yes	1.05 (0.54, 2.04)	0.88		0.93(0.45, 1.91)	0.84
*FGFR* aberration	No	Ref			Ref	
	Yes	1.87 (1.05, 3.33)	0.03		2.17 (1.17, 4.02)	0.01
*NF1* aberration	No	Ref			Ref	
	Yes	1.03 (0.38, 2.81)	0.96		0.6 (0.30, 1.56)	0.37
TMB index	Low	Ref			Ref	
	High	1.94 (1.19, 3.16)	0.008		1.62 (1.00, 2.66)	0.05
	Unknown	0.73 (0.36, 1.47)	0.38		0.88 (0.43, 1.79)	0.72

### KM curves after PMS

Since ER/PR status, *TP53* mutations, *FGFR* aberrations and TMB levels were imbalanced between *PIK3CA*-HD mutant and *PIK3CA*-KD mutant MBC patients, we performed a 1:1 propensity score matching (PSM) strategy to avoid the potential bias. Kaplan-Merier (KM) curves after PSM still showed a marginally significant difference between *PIK3CA*-HD mutant and *PIK3CA*-KD mutant MBCs (Supplementary Figure 1, Logrank p=0.13), suggesting *PIK3CA*-HD mutation itself to be an important risk factor for poor prognosis for MBCs in late-line therapy.

## DISCUSSION

For progressed MBCs after the early-line (≤ 2-line) therapies, there is no consensus on the late-line therapy. Potential choices for hormone receptor (HR)-positive MBCs include mTOR inhibitor, FGFR inhibitor, Estradiol and Progestin [23, 24]. Candidates for metastatic TNBCs might be PD1/PD-L1 inhibitors, anti-VEGFR, etc [25, 26]. Novel TKIs and PD-L1 inhibitors might be useful in HER2-positive patients [27, 28]. In this study, we found that the conception of HR/HER2 status was gradually obscured in late-line therapy. Instead, the genetic aberrations and immune checkpoints became more and more important.

After early-line therapies, MBC tumors become more heterogenous and have more somatic gene aberrations. *PIK3CA*, *ESR1* and *GATA3* mutations increased in progressed patients after chemotherapy [15]. The rate of *ESR1* mutations increased after AI treatment [29]. After the treatment of CDK4/6 inhibitors, *RB1* mutation could be detected by ctDNA testing [30]. Based on these observations, we hypothesize that after multi-line therapies, the genotype of MBC tumors will change significantly, which may lead to the resistance of MBC tumors to the standard therapies that were designed based on HR/HER2 status. In addition, MBC tumors might become “warmer” after multi-line therapies, because the new mutations might generate and present many novel TAAs. Therefore, in terms of late-line therapy, we need to consider more about tumor genotype and immune checkpoints.

In recent years, the usage of antibodies to block the immune checkpoint PD-1 / PD-L1 has become a promising treatment strategy for cancer patients. However, many patients have failed to respond to PD-1 / PD-L1 treatment. Therefore, plenty of researches have focused on the biomarkers to distinguish the responders and non-responders for PD-1/PD-L1 antibody treatment [31]. IMpassion 130 study showed that the low positivity rate of 1% PD-L1 expression rate could be sufficient for patients to have a better response to PD-L1 antibody in triple-negative breast cancers (TNBC) [32]. In addition, a higher non-synonymous mutation or candidate neoantigen burden in tumors (TMB) improved the treatment response of PD-1 antibody in lung cancers [19]. In this study, *PIK3CA*-HD mutations were concentrated in HR-positive patients, and more than half of these patients were TMB-H (Figure 3). Thus, for this group of patients, PD-1 antibody might be a reasonable choice.

In this study, we found that in MBC patients with PI3K/AKT pathway aberration, *TP53*, *ERBB2*, *FAT1*, and *FGFR* aberrations increased significantly (Table 2). In particular, *FGFR* aberration and TMB-high patients concentrated in *PIK3CA*-HD mutant MBC patients, suggesting this specific genotype might be particularly related to poor prognosis and immune checkpoints disruption. This study categorized PI3k/AKT mutations precisely, and clearly identified the specific genotype (*PIK3CA*-HD) for novel treatment strategies, such as PD-1 inhibitors, FGFR inhibitors, etc.

Here, we found that both *PIK3CA*-HD mutations and FGFR aberration seemed to concentrate in HR-positive MBC patients. Is there any potential molecular mechanism underlying the coincidence? However, there is no report about the interaction between *PIK3CA*-HD mutations and FGFR aberration. In FGFR2^mutant^ endometrial cancer, the sub-therapeutic doses of PI3K inhibitors could enhance the efficacy of anti-FGFR therapies [33]. Such a synergic anti-tumor effect suggested *PIK3CA* mutation and *FGFR* aberration might be independent to each other. If they have interaction, inhibiting each of them might also affect the other one. Only when they were independent, PI3K inhibitors and anti-FGFR therapies have synergic effects.

*PIK3CA*-HD mutant MBC patients showed resistance to mTOR inhibitor Everolimus. More than 70% of patients who received Everolimus in late-line therapy progressed within six months. Due to the limited sample number, we did not demonstrate a concrete result here. However, we still suggested that for *PIK3CA*-HD mutant MBC patients, mTOR inhibitor Everolimus might be not as effective as in other PI3K/AKT pathway aberrant patients. PD-1 inhibitor plus FGFR inhibitor might be a promising option.

This study involved 193 MBC patients who received late-line therapies from three affiliated hospitals in Central South University. The limitation was the relatively small sample size for each PI3K/AKT pathway aberrant category. The sample size is even smaller after PSM. In the foreseeable future, we would accumulate many more samples to further validate our hypothesis. For now, we raised our hypothesis and provided clues for reasonable treatment strategies, which might be beneficial for MBC patients who have no choice.

## CONCLUSION

MBC patients with *PIK3CA* mutations in helical domain had a specific ctDNA profile with high TMB and high *FGFR* aberration rate, which might lead to poor PFS for late-line therapy. PD-1/PD-L1 inhibitor and FGFR inhibitor could be promising as a late-line option for MBC patients with this specific genotype.

## MATERIALS AND METHODS

### Patients

This study included MBC patients who were within 2 weeks after progression of the early-line (≤2) salvage treatment. All patients had a confirmed pathology diagnosis (histology type: invasive ductal carcinomas or lobular carcinomas). According to RECIST 1.1 standards, patients had at least one measurable distant disease lesion. All patients had an Eastern Cooperative Oncology Group/World Health Organization Performance Status (ECOG/WHO PS) of 0 or 1. Patients with inflammatory BC, multiple primary malignancies, immunodeficiency or organ transplantation history were excluded. Patients who have received mTOR inhibitor treatment were also not eligible for the study.

### Study design

This multicenter study was conducted across three local cancer centers. MBC patients who failed in early-line (≤2 lines) standard chemotherapies or standard chemotherapies combined with anti-HER2 therapies or endocrine therapies received big-panel NGS for ctDNA testing. After ctDNA testing, patients received late-line therapy.

The primary endpoint was PFS, defined as the number of days from the beginning date of late-line therapy to progressive disease, defined as: 1) >20% growth of measurable target lesions and the absolute increase of target lesions >5mm, or 2) presence of new lesions [34]. There was no predetermined per-patient follow-up period. Patients visited clinics every week. Disease progression was assessed at every visit by imaging tools (MRI, CT, ECT and ultrasound, *etc*) [34].

### ctDNA testing and TMB calculation

Peripheral blood samples were collected within 7 days before treatment. We performed ctDNA testing according to protocol described previously [5, 35, 36]. The main steps included DNA extraction, target capture, NGS and sequencing data analysis. Tumor mutation burden (TMB) is an important prognostic factor [19]. Usually, TMB is calculated from whole exome sequencing data or big gene panels [17, 37]. TMB was determined by analyzing the somatic mutations per mega-base (Mb). TMB analysis interrogated SNVs and small indels with the variant allele frequency (VAF) ≥3%. TMB-U (unknown) is defined as the maximum VAF <3%. A cut-off of the top 25% of the TMB of all BC samples from Geneplus database was 9 mutations (Muts) / Mb. In this study, TMB greater than 9 Muts / Mb was defined as TMB-H (high). TMB less than 9 Muts / Mb was defined as TMB-L (low).

### Statistical analysis

To search for significant ctDNA aberrations, the R package “ComplexHeatmap” was used to rank the hot genetic aberrations in PI3K/AKT pathway aberrant subgroups. A two-sided log-rank test was used to test the influence of PI3K/AKT pathway aberrations in terms of late-line treatment PFS in MBCs. To reduce the potential bias between subgroups, we also performed 1:1 propensity score matching (PSM) analysis as described previously [38]. Both the univariate and multivariate Cox proportional hazards regression analyses were used to evaluate the prognostic factors for PFS. All statistical analyses were conducted by using SAS 9.4 and R 3.6.0 software. All tests of hypotheses were two-tailed and conducted at a significance level of 0.05 and at a marginal significance level of 0.15.

### Ethics

The study protocol and informed consent form were censored by the independent ethics committee for each center. In our study, each participant filled and signed an approved written informed consent provided by the independent ethics committee.

### Statement of translational relevance

12 years ago, Barbareschi M's team reported *PIK3CA* mutations in the helical domain exon 9 were associated with significantly worse prognoses in breast cancer. That study used frozen samples from 163 surgery patients. Here, we demonstrate the effect of *PIK3CA* helic domain mutations in metastatic breast cancer with late-line therapy by using plasma ctDNA. MBC patients with *PIK3CA* mutations in helical domain had a specific ctDNA profile with high TMB and high *FGFR* aberration rate, leading to poor PFS for late-line therapy. Our findings directly reflect the effect of genetic aberrations on treatment outcome and suggest potential strategies for MBC patients. PD-1/PD-L1 inhibitor and FGFR inhibitor could be promising as a late-line option for MBC patients with this specific genotype.

## Supplementary Material

Supplementary Figure 1

Supplementary Table 1
