# Supplementary material for: PIK3CA gene mutations in the helical domain correlate with high tumor mutation burden and poor prognosis in metastatic breast carcinomas with late-line therapies
Source: Aging (Albany NY). 2020 Jan 24;12(2):1577–90. doi: 10.18632/aging.102701 (PMC7053638; doi:10.18632/aging.102701)
Supplement: Supplementary Figure 1 [file aging-12-102701-s002..pdf]

SUPPLEMENTARY FIGURE

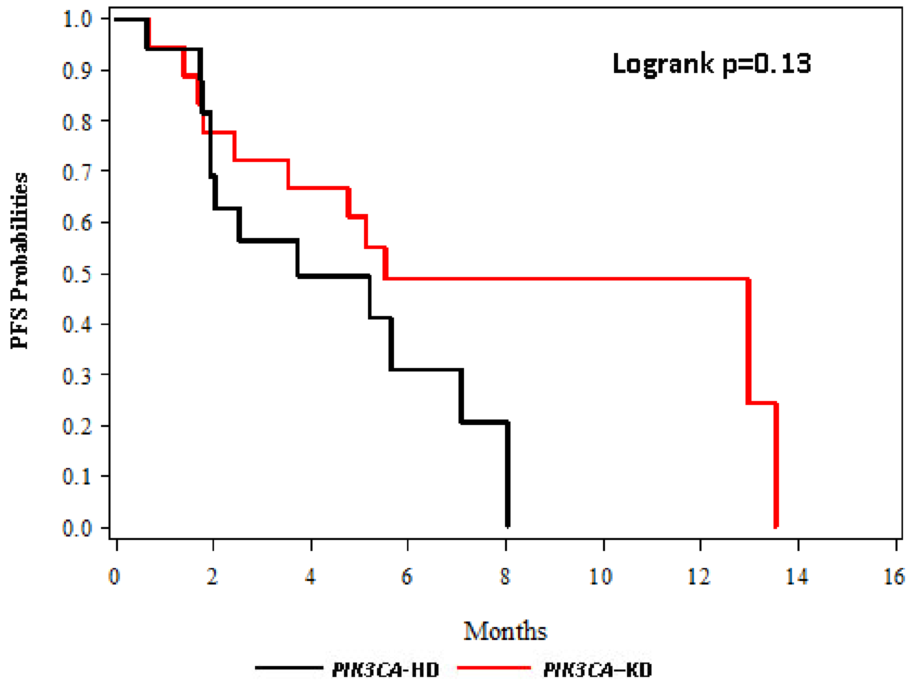

Supplementary Figure 1. KM curves stratified by *PIK3CA*-HD mutations and *PIK3CA*-KD after PMS.
