# Supplementary material for: PIK3CA gene mutations in the helical domain correlate with high tumor mutation burden and poor prognosis in metastatic breast carcinomas with late-line therapies
Source: Aging (Albany NY). 2020 Jan 24;12(2):1577–90. doi: 10.18632/aging.102701 (PMC7053638; doi:10.18632/aging.102701)
Supplement: Supplementary Table 1 [file aging-12-102701-s001..pdf]

SUPPLEMENTARY TABLE

Supplementary Table 1. Univariate Cox regression analyses of clinical risk factors for PFS in MBC patients.

Covariates	Level	Hazard ratio (95% CI)	p-value
Age at diagnosis (of BC)		1.008 (0.986, 1.031)	0.49
Age at brain metastasis (years)		1.011 (0.988, 1.034)	0.36
TTM (years)*		1.010 (0.951, 1.072)	0.75
BC Laterality	Left	Ref	
	Right	0.821 (0.529, 1.275)	0.38
	Double	0.568 (0.138, 2.344)	0.43
ER	Negative	Ref	
	Positive	0.968 (0.635, 1.475)	0.88
PR	Negative	Ref	
	Positive	1.102 (0.723, 1.678)	0.65
HER2	Negative	Ref	
	Positive	0.869 (0.537, 1.404)	0.57
HR/HER2 subtypes	Triple Negative	1.239 (0.755, 2.032)	0.40
	HR+/HER2-	1.024 (0.672, 1.560)	0.91
	HR-/HER2+	0.732 (0.417, 1.285)	0.28
	HR+/HER2+	1.147 (0.552, 2.383)	0.71
Metastasis#	Bone-only	0.892 (0.281, 2.828)	0.85
	Visceral	1.435 (0.861, 2.391)	0.17
	Soft tissue	1.263 (0.748, 2.132)	0.38

Note:
TTM* indicated the time from diagnosis of BC to the diagnosis time of metastasis.
Abbreviation: ER (Estrogen Receptor), PR (Progesterone Receptor), HER2 (Human Epidermal Growth Factor Receptor-2).
